# 

**Published:** 1894-12-29

**Authors:** 


**Notices of Births, Deaths, and Marriages** are inserted in this column at a charge of 2s. 6d. for an announcement not exceeding four lines.

**Notice to Advertisers and Subscribers.**

All Advertisements, Orders for Copies of The Hospital, and Business Communications should be addressed to **The Publisher, NOT TO THE EDITOR**, The Hospital, 428, Strand, W.C.

The Cost of Subscription, post free, is as follows:—Per week, 2½d.; per quarter, 2s. 8d.; per half-year, 5s. 3d.; per annum, 10s. 6d. Foreign:—Per Annum, 12s. 8d.; payable, in all cases, in advance.

### NOTICE TO CORRESPONDENTS.

All MS., Letters, Books for Review, and other matters intended for the Editor should be addressed The Editor, The Lodge, Porchester Square, London, W.

The Editor cannot undertake to return rejected MS., even when accompanied by stamped directed envelope.

**"Burdett's Hospital Annual," 1894.**

Fifth year of publication. 900 pp. Boards, gilt lettering. A most complete guide to British, Colonial, Indian, and American Hospitals, Dispensaries, Nursing and Convalescent Institutions, and Asylums. Price 5s. Published by The Scientific Press (Limited), 428, Strand, W.C.

**NOTICE.—The Index for Vol. XVI. (ending September 24th, 1894) is on sale at the Office of this Journal, Price 2½d., post free.**

### SCRAPS AND GLEANINGS.

The popularity of cremation in Manchester is advancing. Last year 47 persons were cremated.

Smallpox continues prevalent at Edinburgh, and large numbers of persons are daily being vaccinated.

A well-known chemist in Moscow has given a valuable house and £500 to fit it as a bacteriological laboratory.

The governors of the Lunatic Asylum at Dublin have arranged to appoint a lady medical superintendent of the female wards. There are at present upwards of 1,000 patients in the asylum.

A new mission hospital has just been built and presented to the mission station of the Protestant Episcopal Church in Wuch-ang, China, by President Seth Low, of Columbia College, and his brother.

A meeting of the council of the Hospital Saturday Sports Society was held on Wednesday. All clubs had been invited to send representatives as the usual meeting at the Mansion House will not take place this year.

An excellent concert was given on the 13th of this month for the entertainment of the inmates of the National Hospital for Paralysis and Epilepsy. The music was vocal and instrumental, and the evening proved a great success.

Last week the pupils of the School for the Indigent Blind gave a very successful concert. After the concert the various articles manufactured in the school were displayed, and showed excellent examples of really high-class workmanship.

The Board of Managers of the University of Pennsylvania Hospital have decided to set aside certain beds for cases of appendicitis, for the special use of Professors W. Pepper and J. W. White, who are engaged in a special investigation of that disease.

The funds of the Royal Free Hospital have been materially benefited by the ball given in its aid on the 12th of this month. The entertainment was the sixth annual gathering of the kind, and funds have never been more pressingly needed than this year, for the efficient maintenance of the institution.

The Alexandra Hospital for Children is in great financial difficulty, and the committee are making an urgent appeal for help from the charitable public. The total annual expenditure is about £2,500, and the income from investments amounts to about £40, the remainder having to be collected by subscription and donation.

A Lengthy report was read at the annual meeting of the committee of the Children's Hospital of Cold Ash, lately held at Newbury. The balance sheet showed an increase in the subscriptions. The Floral Fête and theatricals for the benefit of the institution realized £87 16s., and there had been several handsome donations during the 12 months under review. A balance remains to the good of £13 17s. 3d., as against a similar sum in deficit at the beginning of the year.

### The Student of Medicine.

### APPOINTMENTS.

W. Anderson, L.R.C.P.Lond., L.S.A., appointed Consulting Surgeon to the Sevenoaks Hospital for Diseases of the Hip. B. Anningson. B.A.Cantab., M.D., M.R C.S., reappointed Medical Officer of Health to the Chesterton Local Board. Dr. J. A. Blayney, appointed Medical Officer and Resident Magistrate in British New Guinea. H. Bullen, L.D.S., appointed Dental Surgeon to the Royal Cornwall Infirmary. H. S. Byers, M.B.Durh., M.R.C.S.Eng., L.R.C.P.Lond., appointed Junior House Surgeon to the Northern Hospital, Liverpool. L. J. G. Carré, M.D.Brux., M.R.C.S., L.R.C.P., appointed Registrar and Anæsthetist at the Royal Hospital for Children and Women, Waterloo Bridge Road. W. Challenor, B.A.Camb., M.R.C.S.Eng., appointed Medical Officer for the Windle District of the Prescot Union. J. Cotton, L.R.C.P.Lond., M.R.C.S.Eng., appointed Medical Officer for the Eccleston District and Public Vaccinator for the St. Helen's District of the Prescot Union. J. Dacre, M.R.C.S., L.R.C.P., reappointed House Surgeon to the Bristol Hospital for Sick Children and Women. H. S. Elliott, M.R.C.S.Eng., L.R.C.P. Lond., L.S.A.Lond., late House Physician at St. George's Hospital, London, appointed House Surgeon to the West London Hospital. H. B. Emerson, M.R C.S.Eng., L.R.C.P.Lond., appointed Visiting Surgeon to the Chester General Infirmary. G. W. Farley, M.B., Ch.B.Roy.Univ.I., appointed House Surgeon to the Bangor Infirmary, North Wales. C. Farrar, M.D.Heidelb., L.F.P.S.Glasg., reappointed Medical Officer of Health to the Chatteris Local Board. J. Galloway, M.D., F.R.C.S.Eng., Assistant Physician to the Great Northern Hospital, appointed Physician in Charge of the Department for Diseases of the Skin, Charing Cross Hospital. F. de Haviland Hall, M.D., F.R.C.P., appointed Physician to the Westminster Hospital, vice Octavius Sturges, M.D., deceased. W. J. Harper, M.R.C.S., L.R.C.P.Lond., appointed Medical Officer for the Braunton District of the Barnstaple Union. G. Home, M.B., C.M.Edin., appointed House Physician to the Northern Hospital, Liverpool. Mr. C. Hood, appointed Medical Officer of Health to the Rural Sanitary District of the Strood Union. A. P. Laurie, M.A., B.Sc., F.R.S.E., late Fellow of King's College, Cambridge, appointed Lecturer on Chemistry and Physics at St. Mary's Hospital Medical School.

A. A. Mussen, A.B., M.B., B.Ch.Dub., appointed Senior House Surgeon to the Northern Hospital, Liverpool. T. Newby, M.D.St.And., M.R.C.S.Eng., reappointed Medical Officer of Health for the Borough of Great Grimsby. J. W. O'Bryen, M.D., L.R.C.P., L.R.C.S., appointed Medical Officer to the Crystal Palace District Gas Company. G. Porter, M.B., C.M. Edin., appointed House Surgeon to Out-patients at the Hospital for Sick Children, Great Ormond Street, W.C. C. G. Roberts, M.A.Camb., M.B., reappointed Medical Officer of Health to the Halstead Local Board. B. Rogers, M.H., B.A., M.D., B.Ch., appointed Pathologist to the Bristol Hospital for Sick Children and Women. W. R. Smith, M.B., B.S.Lond., appointed Medical Registrar to King's College Hospital. J. Somerville, M.D., appointed Medical Officer for the Neepsend District of the Sheffield Union. J. K. Watson, M.B., C.M.Edin., appointed Second Assistant House Surgeon to the Sheffield General Infirmary.

### VACANCIES.

The following vacancies are announced this week, the amount of salary in each case being given after the name of the institution:

### Resident Medical Officers.

Birmingham Midland Free Hospital for Sick Children.—Resident Medical Officer and Resident Surgical Officer. Salary, £70 and £50 per annum, respectively, with board, washing, and attendance in the institution. Applications to the Secretary, Children's Hospital, Steelhouse Lane, Birmingham, January 8th.

Devon and Exeter Hospital, Exeter.—Assistant House Surgeon; doubly qualified, and unmarried Salary, £40 per annum, with board and lodging. Applications to Albert E. Boyce, Secretary, by January 7th.

Norfolk and Norwich Hospital.—Assistant to the House Surgeon, doubly qualified. Appointment for six months. Board, lodging, and washing provided. Applications to the House Surgeon by January 5th.

West Herts Infirmary, Hemel Hempstead.—House Surgeon and Dispenser, doubly qualified and unmarried. Appointment for two years. Salary, £100 per annum, with board, furnished rooms, fire, lights, attendance, and washing. Applications to the Secretary by January 17th.

York County Hospital.—Assistant House Surgeon; doubly qualified. Salary, £60 per annum, with board, rooms, washing, and attendance. Applications to C. E. Pinfold, Secretary, by January 3rd.

### Non-Resident Medical Officers.

Chelsea, Belgrave, and Brompton Dispensary, 41, Sloane Square, S,W.—Honorary Physician. Applications to the Secretary by Jannar 3rd.

Chelsea Hospital for Women, Fulham Road, S.W.—Two Physicians to In-patients, one Surgeon to In-patients, three Physicians to Out-patients, one Surgeon to Out-patients, a Pathologist, and a Registrar. Must be engaged in consulting practice only. Applications, on forms to be obtained of the Secretary, to be sent in by January 4th.

Eltham College, Kent (Royal Naval School).—Medical Officer, Applications to the Rev. the Bursar by January 5th.

Hospital for Sick Children, Great Ormond Street, W.C.—Two Assistant Physicians; must be F. or M.R.C.P Lond. Applications to the Secretary by January 4th.

London County Council.—Pathologist to the London County Asylum. Salary, £700 per annum, with travelling expenses. Applications, on forms to be obtained at the office of the Committee, endorsed "Application for Pathologist," to R. W. Partridge. Clerk to the Asylums Committee, London Asylums Committee Office, 21, Whitehall Place, S.W., by Ten o'clock on January 5th.

Westminster Hospital. — Fourth Assistant Surgeon. Must be F.R.C.S.Eng. Candidates must transmit certificate of age. and attend the House Committee on January 1st.

### THE NURSES' BOOKSHELF.

**NURSING.** By Isabel Adams Hampton. 2nd Edition, 500 pp., Seven plates. Eighteen illustrations, &c., 7s. 6d. net.

**HOSPITAL SISTERS AND THEIR DUTIES.** By Eva C. E. Luckes. Second Edition. 2s. 6d.

**HOW TO BECOME A NURSE.** By Honnor Morten. Illustrated. Price 2s. 6d.

**"THE HOSPITAL" NURSE'S CASE-BOOK.** Demy 16mo. (for the apron pocket), 72 pp., strong boards. Second Edition, price 6d., or 4s. 6d. per dozen, post free.

A Book of Tables specially prepared for use by Nurses in the Ward and in the Sick-room. Containing Twenty-six complete Tables, each being ruled to last one week, for keeping an exact record of a patient's condition, including notes on Temperature, Pulse, Respiration, Motions, Age, Sex, Name, Address, Occupation, Treatment, History, Name of Medical Attendant, Number of Case, and Date of Admission.

Nurses should combine together and order parcels of over a dozen to profit by the special discount terms—*i.e.*, 4s. 6d. per dozen, post free.

**SURGICAL WARD-WORK.** A Practical Manual of Clinical Instruction for Students and Nurses. By ALEXANDER MILES, M.D.Edin., C.M., F.R.C.S.E. 199 Illustrations, demy 8vo., 200 pp. Price 3s. 6d.

**OPHTHALMIC NURSING.** By Sydney Stephenson. 200 pp. Illus., 3s. 6d. With Glossary of Terms, Lists of Instruments, &c.

**MENTAL NURSING.** By William Harding, M.D. Second and Revised Edition 2s. 6d.

**ART OF MASSAGE.** By A. Creighton Hale. Seventy Illustrations. 6s.

**OUTLINES OF INSANITY.** By Francis H. Walmsley, M.D. Popular Edition. 1s. 6d.

**ART OF FEEDING THE INVALID.** By A Medical Practitioner and a Lady Professor of Cookery. 3s. 6d.

**THE MOTHER'S HELP,** and Guide to the Domestic Management of Children. By P. MURRAY BRAIDWOOD, M.D. Cloth gilt, 140 pp. Price 2s. 6d.

